# Personalized Analytics and a Wearable Biosensor Platform for Early Detection of COVID-19 Decompensation (DeCODe): Protocol for the Development of the COVID-19 Decompensation Index

**DOI:** 10.2196/27271

**Published:** 2021-05-26

**Authors:** Karen Larimer, Stephan Wegerich, Joel Splan, David Chestek, Heather Prendergast, Terry Vanden Hoek

**Affiliations:** 1 physIQ, Inc Chicago, IL United States; 2 Emergency Department University of Illinois Health Chicago, IL United States

**Keywords:** analytic, artificial intelligence, biomarker, cloud, COVID-19, decompensation, detection, development, index, monitoring, outcome, remote monitoring, symptom validation, wearable

## Abstract

**Background:**

During the COVID-19 pandemic, novel digital health technologies have the potential to improve our understanding of SARS-CoV-2 and COVID-19, improve care delivery, and produce better health outcomes. The National Institutes of Health called on digital health leaders to contribute to a high-quality data repository that will support researchers to make discoveries that are otherwise not possible with small, limited data sets.

**Objective:**

To this end, we seek to develop a COVID-19 digital biomarker for early detection of physiological exacerbation or decompensation. We propose the development and validation of a COVID-19 decompensation Index (CDI) in a 2-phase study that builds on existing wearable biosensor-derived analytics generated by physIQ’s end-to-end cloud platform for continuous physiological monitoring with wearable biosensors. This effort serves to achieve two primary objectives: (1) to collect adequate data to help develop the CDI and (2) to collect rich deidentified clinical data correlating with outcomes and symptoms related to COVID-19 progression. Our secondary objectives include evaluation of the feasibility and usability of pinpointIQ, a digital platform through which data are gathered, analyzed, and displayed.

**Methods:**

This is a prospective, nonrandomized, open-label, 2-phase study. Phase I will involve data collection for the digital data hub of the National Institutes of Health as well as data to support the preliminary development of the CDI. Phase II will involve data collection for the hub and contribute to continued refinement and validation of the CDI. While this study will focus on the development of a CDI, the digital platform will also be evaluated for feasibility and usability while clinicians deliver care to continuously monitored patients enrolled in the study.

**Results:**

Our target CDI will be a binary classifier trained to distinguish participants with and those without decompensation. The primary performance metric for CDI will be the area under the receiver operating characteristic curve with a minimum performance criterion of ≥0.75 (α=.05; power [1–β]=0.80). Furthermore, we will determine the sex or gender and race or ethnicity of the participants, which would account for differences in the CDI performance, as well as the lead time—time to predict decompensation—and its relationship with the ultimate disease severity based on the World Health Organization COVID-19 ordinal scale.

**Conclusions:**

Using machine learning techniques on a large data set of patients with COVID-19 could provide valuable insights into the pathophysiology of COVID-19 and a digital biomarker for COVID-19 decompensation. Through this study, we intend to develop a tool that can uniquely reflect physiological data of a diverse population and contribute to high-quality data that will help researchers better understand COVID-19.

**Trial Registration:**

ClinicalTrials.gov NCT04575532; https://www.clinicaltrials.gov/ct2/show/NCT04575532

**International Registered Report Identifier (IRRID):**

DERR1-10.2196/27271

## Introduction

During the COVID-19 pandemic, novel digital health technologies have the potential to improve our understanding of SARS-CoV-2, improve health care delivery, and produce better outcomes. In addition to digital technologies, there is a need for a high-quality COVID-19 research data repository that allows academic, public health, and translational researchers to make discoveries that might otherwise not be possible from data silos. Given the breadth and specialization of digital technologies that will be utilized during the COVID-19 pandemic, it is likely that there are research questions that can only be answered by integrating and analyzing data generated through multiple technologies. The National Institute of Biomedical Imaging and Bioengineering and National Cancer Institute of the National Institutes of Health (NIH) issued a request for proposals and awarded contracts [1] for the development of digital health solutions to address the COVID-19 pandemic by enabling new studies on these technologies. Data generated by digital health technologies could potentially advance the public health response, facilitating underlying approaches that might improve planning for future epidemics and pandemics. 

In response to the request for proposals, physIQ collaborated with the NIH to deploy a method of collecting an immense volume of physiological data on patients with COVID-19 and develop a COVID-19 biomarker or index that could facilitate early detection of decompensation. In other words, the goal is to identify when an individual starts transitioning from being SARS-CoV-2–positive to having acute COVID-19. Such early identification would allow clinicians to intervene early and possibly prevent acute clinical events including hospitalization. This study aims to develop and validate a COVID-19 decompensation index (CDI) in a 2-phase manner and build on existing wearable biosensor–derived analytics generated by physIQ’s end-to-end cloud platform for continuous physiological monitoring with wearable biosensors.

The development of a CDI is believed to be key to managing and mitigating the severity of a patient’s illness, especially COVID-19 where little is known about the progression of the disease. Using an easy-to-wear biosensor (patch), which provides streamed physiological data for analysis by state-of-the-art analytics, could yield significant advantages during in-person, telephonic, or survey-based patient assessments, which are the basis for remote patient monitoring solutions today [2]. Most remote patient monitoring solutions have yet to harness recent advancements in biosensor devices and machine learning technologies and thus do not allow for intelligent continuous patient monitoring. Furthermore, any data that are collected from patients are typically funneled through already overburdened clinical providers. This may be the reason why most remote monitoring solutions for COVID-19 are limited to temperature and pulse oximetry spot checks. In fact, there is no evidence that such spot checks are an efficacious strategy; rather, they are used only because they are familiar variables to health care professionals and are sampled at a manageable frequency [3]. This is especially true considering that previous studies suggest that <50% of hospitalized patients with COVID-19 have a fever upon admission [4,5], and studies on other viral infections have reported that a significant increase in heart rate can be detected approximately 2 days prior to a fever [6]. Early warning signs of COVID-19 are probably being missed because of focusing only on limited spot checks rather than early changes in the overall cardiopulmonary status, as reflected by the combination of variables such as respiration rate, heart rate, heart rate variability, physical activity levels, and other metrics that can be derived from biosensor data [7].

The equitable deployment of digital health solutions is equally as important as developing a digital biomarker or index. Populations that are underserved in health care also have less access to advanced digital health solutions owing to various social determinants of health [8]. The development of a tool that can uniquely address a barrier to care, reflect the physiological data of diverse populations, and contribute to a trove of high-quality data that would help researchers in better understanding COVID-19 in all populations is of paramount importance [9].

While this study is exploratory, we expect parameters including respiratory rate and heart rate to be predictive of decompensation, considering that they may vary in the degree to which they are predictive for any individual. We hypothesize that a combination of many biosensor-derived physiological features will best capture the heterogenous characteristics of decompensation across the population. Our digital biomarker development approach will harness the availability of a large number of continuous physiological features that characterize all aspects of physical function and can reflect decompensation (heart rate, respiratory rate, heart rate variability, activity, sleep, arrhythmias, skin temperature, and others).

Our study responds to the need for novel technologies to manage the COVID-19 pandemic and aims to develop and validate a CDI by using physIQ’s pinpointIQ platform in a diverse population of adults with COVID-19. This effort will achieve two primary objectives: (1) to collect adequate data to enable the development of the CDI and (2) to collect rich deidentified clinical data correlating with outcomes and symptoms related to COVID-19 progression. Our secondary objectives include the evaluation of feasibility and usability of pinpointIQ, the digital platform on which data are gathered, analyzed, and displayed.

## Methods

### Study Design

This is a prospective, nonrandomized, open-label, 2-phase study. This study design was chosen because the primary focus of the study, in addition to data collection for the NIH digital data hub, is the development of a digital biomarker for early detection of COVID-19 decompensation. This initially requires the determination of the model structure and predictor variables (phase I), followed by validation and performance assessments of the CDI (phase II). Phase I will involve data collection for the NIH digital data hub as well as the collection of data to support the preliminary development of the CDI. In doing so, “decompensation events” will be identified during the 28-day participant monitoring period. A “decompensation event” is defined as a hospitalization event due to COVID-19 during which a patient achieves a maximum World Health Organization ordinal scale for clinical improvement (WHO OSCI) score of ≥3 [10]. A WHO OSCI score of 3 corresponds to “hospitalization, no oxygen therapy” [10]. Health records will be obtained from the care facility on any clinical event for final adjudication as a COVID-19 decompensation event. Once adjudicated, the event is added to the superset of decompensation events to drive the development of a CDI model. Once a critical number of decompensation events occur in the study sample, the event and nonevent data will be combined and partitioned into training and testing subsets to enable the development of the initial CDI.

Phase II will also involve data collection for the NIH digital data hub and will contribute to continued refinement and validation of the CDI as additional decompensation events are recorded. While this study will focus on the development of a CDI, the pinpointIQ platform will also be evaluated for feasibility and usability while clinicians deliver care to continuously monitored patients enrolled in the study.

### Participants

Participants will be recruited from two pools of patients at University of Illinois Health: (1) patients who test positive for COVID-19 in an outpatient setting and (2) patients who were hospitalized with a diagnosis of COVID-19 and subsequently discharged for home convalescence. This will yield a convenience sample. To be enrolled in the study, patients must meet the following eligibility criteria: they must be aged ≥18 years, test positive for COVID-19, be able to respond manually to a survey on a provided smartphone, and complete the informed consent process. Participants would then be enrolled in the pinpointIQ could platform and trained on the system.

### Data Collection

**Figure 1:**
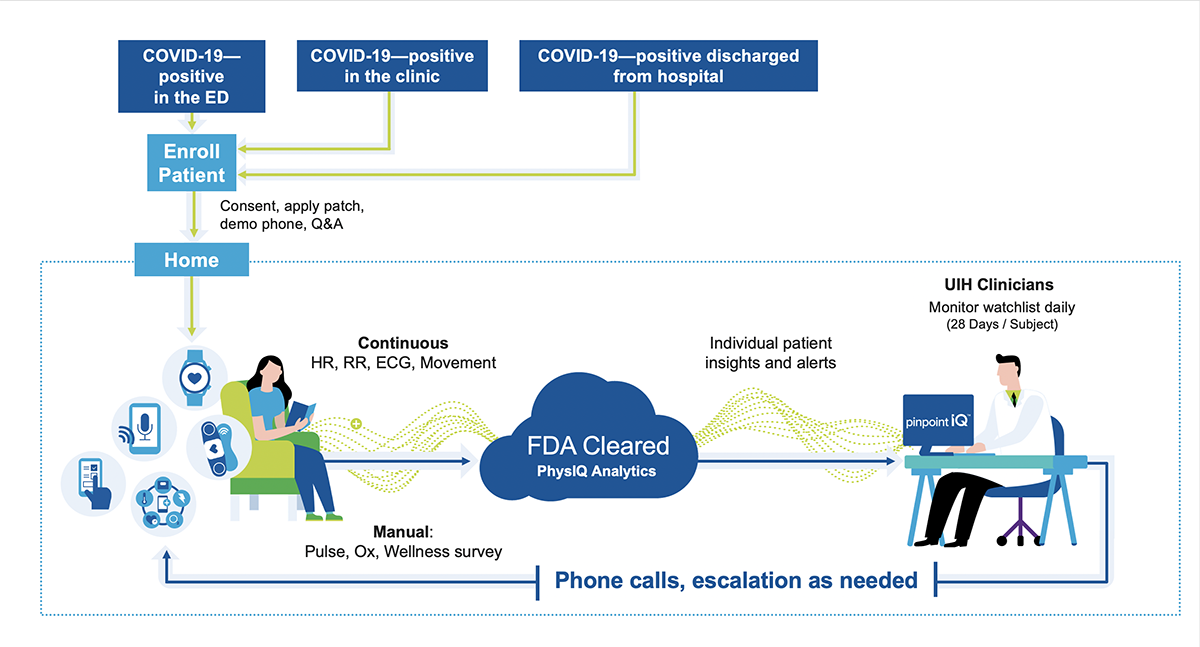
Study protocol. ED: emergency department, HR: heart rate, RR: respiratory rate, ECG: electrocardiography, UIH: University of Illinois Health. ©2021 physIQ.

Upon enrollment, the participant will be shipped a kit that includes biosensor patches, a locked Android smartphone, a charger, pulse oximeter, and a quick start Guide. Participants are remotely enrolled through electronic consent through a trial management platform. The research associate trains the participant on patch application and the phone app survey response. The patient wears the patch for 28 consecutive days at 5-7–day intervals (corresponding to the battery life of the biosensor). Participants can independently change their chest-worn patch. Once the participant has the biosensor applied and is enrolled on the platform, physiological data will immediately begin streaming to the cloud for analytic (applied machine learning) purposes and for clinical monitoring (Figure 1). The pinpointIQ platform is a secure, scalable, device-agnostic, cloud-based software product. The platform performs continuous collection and processing of high-fidelity physiological data.

This continuous patient remote monitoring platform, pinpointIQ, includes Food and Drug Administration (FDA)–cleared analytics that can provide early indications of nonspecific patient deterioration, facilitate data collection, and notify general clinician-defined events. This closed-loop monitoring platform is comprised of the following components, each of which will be used in this study.

The biosensor is VitalPatch (VitalConnect Inc), which is an FDA-approved wireless, battery-operated wearable biosensor worn on the torso to acquire 125-Hz electrocardiography signals, 0.25-Hz impedance, 50-Hz triaxial accelerometric measurements, and 0.25-Hz skin temperature data. A library of analytics on the pinpointIQ platform is applied to the raw sensor data to derive a collection of vital signs and physiological features, including heart rate, respiration rate, heart rate variability, activity level, sleep-wake determination, and atrial fibrillation detection.

**Figure 2:**
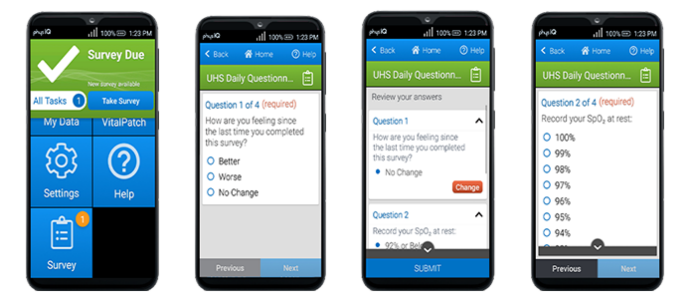
Smartphone app and an example survey.

A smartphone is provided to the participant. PhysIQ developed an Android smartphone app that serves as the gateway for real-time data acquisition via bluetooth from the biosensor. It also provides an interface for patient-reported outcome surveys (Figure 2). The app runs on a dedicated locked smartphone that is configured only to run the app, which has been validated to reliably interface and collect data from the biosensor. The app interacts with the biosensors in real time and uploads data directly to the pinpointIQ platform over a secure cellular network connection. The app also provides patients with indications for proper data collection and escalating alerts of potential data lost, including connectivity issues, low battery, low memory, and unanswered surveys.

**Figure 3:**
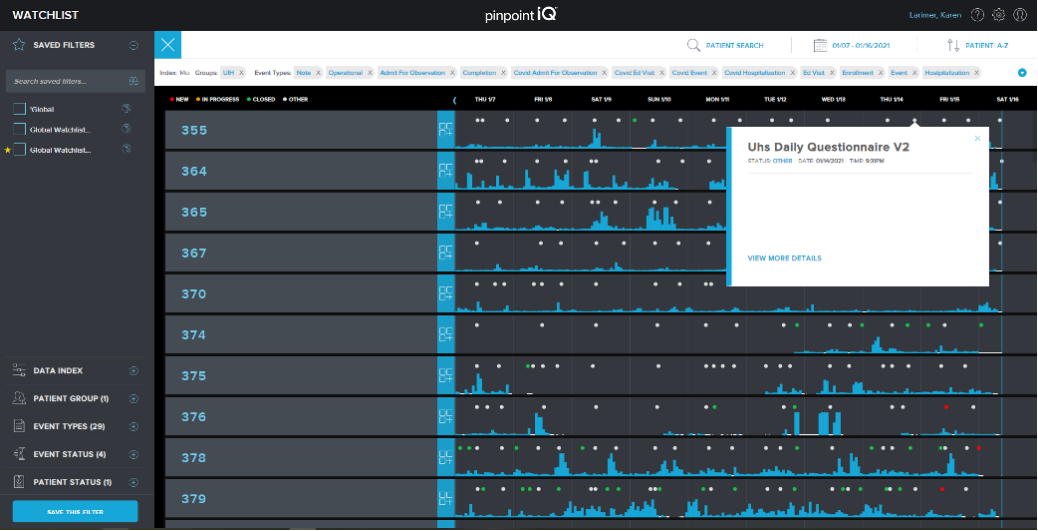
Daily patient reported-health surveys displayed to the clinical user.

To ensure consistent gathering of patient-reported outcome data and spot check peripheral oxygen saturation measurements, digital surveys with health status questions and a reminder to spot check pulse oximetry measurements will be provided to the participants twice daily. Responses will be manually entered into the smartphone app by the participant and be presented as a response in the portal watchlist to the health care professional. While not the primary physiological indicators in this study, these data will enrich and annotate the continuous physiological data collected from the biosensor. These responses are automatically uploaded to pinpointIQ and are immediately flagged as complete on the participant’s dashboard (Figure 3).

Two FDA-approved analytics modules will also be used. The first one is the physIQ heart rate and respiration module, which is a cloud-computing, all-software product that utilizes biosensor input data to derive the heart rate, heart rate variability, and respiration rate. In addition, the heart rate monitoring module includes an atrial fibrillation detection output. The second one is the physIQ personalized physiology analytics module, which is a cloud-computing, all-software product that utilizes biosensor data to compute a multivariate change index (MCI). The MCI is a multiparameter algorithm that establishes a personalized physiological baseline for each patient and monitors for subtle changes from that baseline over time. While the MCI is cleared for subtle physiological changes, we aim to develop an index specifically for the early detection of COVID-19 decompensation (ie, the CDI).

Additionally, the clinical portal component of pinpointIQ provides tools for viewing biosensor data and analytics results, which also drives clinician-defined events as well as application programming interface and software development kit tools for accessing and exporting data and analytics data for research and analysis. The clinical portal and alerts rules engine provide a web-based user interface portal to manage and monitor a specific study or patient population.

**Figure 4:**
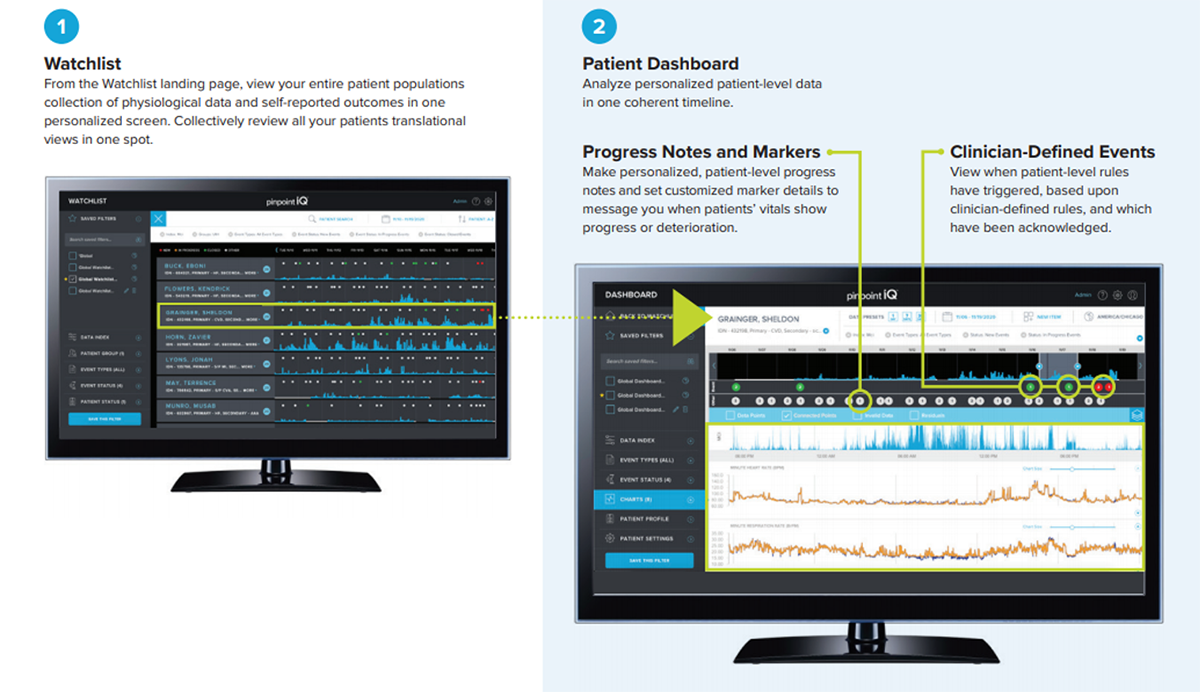
Watchlist and patient dashboard.

Clinicians can review a list of all patients using the watchlist view of the secure, web-based portal. As illustrated in Figure 4, the watchlist can be filtered by groups, patient status, and specific patient identifiers. In addition, the portal allows the assignment of devices (phones and sensors), scheduling of surveys, and modification of patient profiles. Starting from the watchlist, clinicians can access specific patient records on the patient dashboard to examine data in detail, including patient responses to surveys, point measurement values, data records on physiological features, raw biosensor data (eg, electrocardiograms and accelerometry measurements), markers, and clinician notes.

“Clinician-defined events” are also displayed as markers on the watchlist for quick review by clinical users. Clinician-defined events are driven by rules that target specific changes in one or more physiological features over time. Defining rules that would trigger events for participants allows clinical users to identify patients who need attention. PinpointIQ’s rules engine ingests physiological feature data and applies clinician-defined thresholds and persistence criteria to trigger “events” that warrant clinician review or response. Clinician-defined events include, for example, tachycardia, tachypnea, bradycardia, and atrial fibrillation.

**Figure 5:**
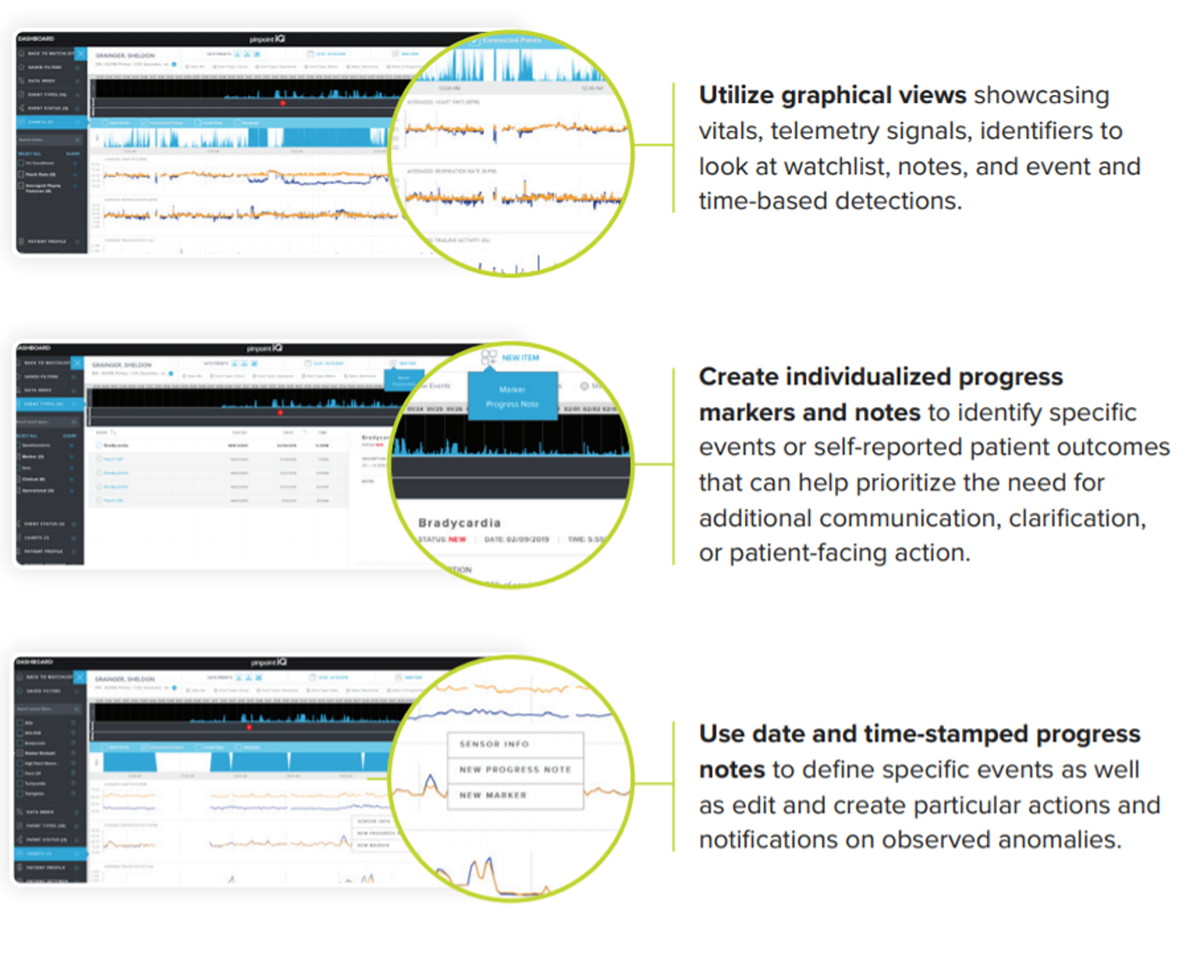
Clinical user's patient dashboard.

Individual patient dashboards would clinicians to view continuously acquired biosensor data from each participant, as well as the occurrence of autogenerated clinician-defined events and clinical notes entered by clinicians over time (Figure 5).

For the target study population, physIQ will apply the full suite of clinical and operational rules currently in production. These rules have a basic template with validated thresholds but can be modified on the basis of clinician needs. The combination of rule outputs, physiological features, and clinical data (spot check measurements, survey results, and demographics) will drive the development of CDI.

### Study Outcomes

Our target CDI will be based on a machine learning model to distinguish between participants with and those without a decompensation event. The primary performance metric for CDI will be the area under the receiver operating characteristic curve (AUC) with a minimum AUC performance criterion of ≥0.75 (α=.05; power [1–β]=0.80). 

The secondary outcomes will include the evaluation of the feasibility of using the pinpointIQ solution as a tool for health care professionals to determine when a participant undergoes decompensation (acute COVID-19) and manage the study populations on the basis of physIQ-validated rule sets and analytics. Feasibility and usability are the primary constructs that will guide the secondary outcomes. Feasibility will be assessed by evaluating the degree to which the monitoring program could be delivered to the participants, assessment of the intended effects of the program on participant outcomes, and the degree to which this program could be utilized with existing means [11].

Overall clinical usability will be determined on the basis of a combination of survey compliance, biosensor data quality, and data availability metrics, as well as load on clinical resources. Survey compliance will be measured by determining the proportion of completed electronic patient-reported outcome surveys and pulse oximetry measurements, obtained using the smartphone app, in accordance with the study protocol. Biosensor data quality assessments will be made using a signal quality index, which would indicate when the acquired biosensor data are usable for deriving accurate physiological features. Data availability will be measured using a biosensor-based “pseudo-compliance” approach that will determine the percentage of data collected relative to the theoretical amount of data that could be acquired. This will be carried out using the first and last timestamps extracted from acquired biosensor data to define the theoretical amount of data that could be collected continuously and then calculating the ratio of the actual amount of data collected to the theoretical amount.

Furthermore, we shall obtain data on participant sex or gender and race or ethnicity, which could impact differences in the CDI performance, as well as the lead time—time to predict physiologic decompensation due to COVID-19—and its relationship with the ultimate disease severity in accordance with the WHO OSCI [10].

### Sample Size and Statistical Analysis

In phase I, we will combine physIQ’s existing personalized analytics, biosensor-derived analytics or aggregates, and clinical rules as input variables to derive the CDI. Cases of COVID-19 decompensation recorded in phase I will inform the final set of input variables to derive the CDI. Our goal is to develop a CDI that relies solely on biosensor-derived physiological features. However, readily available fixed variables such as gender, age, and weight will be examined to determine their impact on the performance of the CDI model. The full set of biosensor-derived features that we intend to analyze as input variables to CDI will be derived from continuous 1-minute measurements of heart rate, respiratory rate, heart rate variability, sleep-wake determination, step rates, activity levels, and other outputs of physIQ’s personalized physiology analytics algorithm.

On conclusion of phase I, all COVID-19 decompensation events (positive) and nonevents (negative) will be combined to assess the performance of the CDI model using the AUC as the performance metric. 

A “clinical event” is defined as an escalation of care from home-based remote monitoring to a higher level of care. For example, if during monitoring, a nurse identifies that a patient is becoming sicker and needs acute care, this event would be considered a clinical event. Health records will be gathered from the care facility on any clinical event for final adjudication as a COVID-19 clinical event. The adjudication consists of review of the electronic health record of the participant experiencing the clinical event and 2 emergency department physicians independently deciding on whether the event was a “COVID-19 clinical event” or “non–COVID-19 clinical event.” If the physicians’ opinions do not agree, the case is further reviewed by a third emergency department physician, and a final decision is made. A COVID-19 clinical event is a “decompensation event” if a patient achieves a maximum WHO OSCI score of ≥3 while hospitalized.

We assume an event rate of 7.5% on the basis of recent readmission rates for patients with COVID-19. To achieve a target CDI performance of an AUC of ≥0.75 with α=.05 and power (1–β)=0.80, we require a sample of 12 positive cases and 148 negative cases for a total sample size of 160 cases. Our proposed sample size of 400 cases in phase I will enable us to partition the data into development (for training) and holdout (for performance assessment) subsets. We intend to use 60:40 partitioning for training and testing, which amounts to 18 positive cases and 222 negative cases for training the CDI model, and 12 positive cases and 148 negative cases for assessing the performance of the CDI model. We anticipate utilizing more events than the minimum required number for performance measures such that heterogeneous COVID-19 decompensation characteristics, as reflected in biosensor-derived analytics, are captured.

The CDI developed in phase I will be deployed in phase II. Based on the performance in phase I, a decision threshold will be defined for use during the deployment of the CDI model in phase II. On conclusion of phase II, the final COVID-19 decompensation event detection performance of the CDI will be validated through secondary AUC analysis. Additionally, exploratory analysis of both lead time to COVID-19 decompensation-related hospitalizations and predictivity of the severity of the decompensation event will be performed. Event time will be defined as the admission date, and severity measures will be based on the WHO OSCI score.

Overall CDI decompensation event detection performance and lead time statistics will be examined across the study population as well as within subgroups. The data will be stratified on the basis of sex or gender and race or ethnicity into subgroups to determine if there are differences in performance and detection lead times among the groups. A sample size of 1200 cases has been chosen for phase II to ensure the capture of data across a diverse population on the basis of known COVID-19–related hospitalization rates for the sex or gender and race or ethnicity subgroups of interest.

### Data Management

After enrollment, each participant will be assigned a unique identifier to be used in the platform. Data will be entered by research staff and clinicians, and data accuracy will be verified by the principal investigator. Data quality control measures include queries to identify missing data and the assessment of outliers and discrepancies. All databases are password-protected. Other cloud security measures include role-based access controls for all URL routes in the application programming interface and within the platform. Patients who withdraw from the study will no longer be monitored and will resume usual care.

Owing to the minimal-risk nature of the study, no external observational study monitoring board is required. The principal investigator and research staff will monitor data internally and meet weekly to ensure the study is proceeding as intended.

## Results

Data collection for phase I has been concluded in end-January 2021 with a preliminary analysis. COVID-19 events captured in phase I will inform the final set of input variables to determine the CDI. Data collection for phase II will begin immediately with anticipated completion in September 2021. We anticipate the completion of study data analysis in November 2021. The final results will be disseminated through scientific publications. Data will also become available through the NIH.

The study protocol has been approved by the institutional review board of the University of Illinois, Chicago. Written informed consent will be obtained from all study participants by the study staff responsible for recruitment. Important protocol modifications will be conveyed to investigators, the institutional review board, regulators, journals, and trial participants. Participant identities will not be disclosed for any public purpose or for publication, nor will they be disseminated outside of the study team.

## Discussion

There is a need for a high-quality COVID-19 research data set that can allow academic, public health, and translational researchers to make discoveries that might otherwise not be possible with small, limited data sets. Novel digital technologies should be leveraged to provide scalable, robust platforms to gather and analyze data quickly and efficiently. The use of machine learning techniques on a large data set of patients with COVID-19 can provide valuable insights into the pathophysiology of COVID-19 and reveal a digital biomarker for physiological decompensation due to COVID-19.

In addition, the highly communicable nature of COVID-19 demands a remote patient monitoring solution. While remote patient monitoring programs for various diseases have been evaluated over the years and have yielded mixed results, the literature demonstrates the lack of consistent positive findings, which leave potential users uncertain of their value [12]. Nonetheless, the demands emerging due to COVID-19 on our health system’s resources require a solution that is scalable and designed to protect clinicians. Through this study, we plan to develop a tool that can uniquely reflect the physiological data of a diverse population and contribute to a trove of high-quality data that will help researchers better understand COVID-19.

## Abbreviations

AUC: area under the receiver operating characteristic curve
CDI: COVID-19 decompensation index
FDA: Food and Drug Administration
MCI: multivariate change index
NIH: National Institutes of Health
WHO OSCI: World Health Organization ordinal scale for clinical improvement
